# More utilitarian judgment in Internet addiction? An exploration using process dissociation and the CNI model

**DOI:** 10.1002/brb3.2510

**Published:** 2022-02-03

**Authors:** Jianxia Lu, Junjie Xie, Jin Chen, Yan Zeng, Zhongli Jiang, Yunqiang Wang, Hui Zheng

**Affiliations:** ^1^ Department of Rehabilitation Medicine The First Affiliated Hospital of Nanjing Medical University Nanjing China; ^2^ School of Rehabilitation Jiangsu Vocational College of Medicine Yancheng China; ^3^ School of Psychology Nanjing Normal University Nanjing China; ^4^ Research Center of Brain and Cognitive Neuroscience Liaoning Normal University Dalian China; ^5^ Shanghai Key Laboratory of Psychotic Disorders Shanghai Mental Health Center Shanghai Jiao Tong University School of Medicine Shanghai China

**Keywords:** CNI model, deontology, Internet addiction, moral judgment, process dissociation, utilitarianism

## Abstract

**Background:**

Internet addiction (IA), which is disadvantageous for decision making, such as moral judgment, is a pernicious threat to contemporary societies. However, few studies consider social cognition abilities as an important variable in IA.

**Objectives:**

This study explores the psychological mechanism of IA facing the moral dilemma.

**Methods:**

Forty participants with IA and 89 healthy participants were recruited. They finished the Internet Addiction Test and completed the moral judgment task. The process dissociation (PD) method and the consequences, norms, and generalized inaction (CNI) model were used to analyze moral judgment data.

**Results:**

Compared with the healthy control (HC) group, the traditional analysis showed that the IA group made more utilitarian judgment regarding moral dilemmas. PD analysis showed that the IA group had decreased deontological inclination, without utilitarian inclination. The CNI model further showed that the sensitivity of the IA group to moral rules was significantly lower than that of the HC group, while there was no significant difference between groups in the sensitivity to the consequences and the general preference for action.

**Conclusions:**

Individuals with IA make more utilitarian judgment when faced with a moral dilemma, which is related to their weak sensitivity to moral norms.

## Graphical abstract

Patients with Internet addiction (IA) made more utilitarian judgment regarding moral dilemmas. Process dissociation analysis showed that the IA had decreased deontological inclination. The CNI model showed the IA had lower sensitivity to moral rules.

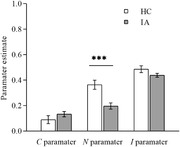

## 1 INTRODUCTION

The number of Internet users has increased rapidly, and the Internet has become an indispensable part of contemporary life (Cerniglia et al., 2017). Rationally using the Internet is beneficial; however, excessive or unlimited use of the Internet may lead to Internet addiction (IA), also known as problematic Internet use (Guo et al., 2018), which may not only damage individuals’ physical and mental health (El Asam et al., 2019) but also have a serious impact on learning, work, and social functions (Duong et al., 2020). Similar to other types of addiction, disadvantageous decision‐making is a core symptom of IA, such as neglecting serious negative consequences and repeating addiction behavior (Ko et al., 2010). As a special decision‐making process (Verdejo‐Garcia et al., 2014), moral judgment often involves from evaluating the right and wrong of behaviors to inferring a person's moral character, or the process of weighing and choosing between different moral principles (Conway & Gawronski, 2013; Malle, 2021).

Moral judgment evaluates the acceptability of behavior according to the concept of right and wrong formed by society (Moll et al., 2005). The sacrificial hypothetical moral dilemma is often used to assess moral judgment, which leads to the opposition between one moral principle and another (Crone & Laham, 2017; Gawronski & Beer, 2017). A typical example is the trolley problem: a runaway trolley is about to crash into five workers (Greene et al., 2001). Should people press the switch to turn the train to another track, sacrificing one innocent person to save five others (Malle, 2021)? Under the utilitarian principle, it is acceptable to sacrifice one innocent person to save five others because doing so can maximize the overall well‐being; however, under the principle of deontology, the sacrifice of an innocent person is a serious violation of moral rules (Conway & Gawronski, 2013). When the individual's judgment in moral dilemma conforms to the utilitarian principle, it is called utilitarian judgment and conforms to the deontological principle, it is called deontological judgment (Reynolds & Conway, 2018).

Why do individuals make utilitarian judgment or deontological judgment? The dual‐process model of moral judgment explains psychological mechanism when dealing with the sacrificial moral dilemma. The moral judgment is driven by two independent and competitive processes, in which utilitarian judgment is driven by cognitive process, while deontological judgment is driven by emotion process (Greene, 2007). According to the traditional analysis of moral dilemma, when an individual accepts the action of pressing the switch, this decision is considered utilitarian judgment; in contrast, saving the innocent person is seen as deontological judgment (Gawronski & Beer, 2017). In this case, however, the two principles are completely negatively correlated. If so, there is no conflict between the two principles in this situation, and the moral dilemma cannot pose a dilemma to the choosing individual (Conway & Gawronski, 2013). In fact, there are still deontological considerations in utilitarian judgment. Relatively stronger utilitarian inclination or weaker deontological inclination may lead to utilitarian judgment (Conway & Gawronski, 2013). The drawbacks of traditional analysis might equate explicit moral judgment with implicit moral inclinations (for example, equating utilitarian judgment with utilitarian inclination) (Brannon et al., 2019).

The new paradigms of moral dilemma are helpful to reveal the deeper level of moral judgment. The process dissociation (PD) method and multinomial processing tree (MPT) were introduced to further clarify the psychological mechanism underlying explicit moral judgment (Gawronski, Armstrong, Conway et al., 2017). The PD analysis involves two versions of the moral dilemma: congruent and incongruent (the latter version would make deontological and utilitarian inclination opposite, while the former does not) (Conway & Gawronski, 2013). By calculating an individual's response to the two dilemmas, the PD method can separate utilitarian inclination from deontological inclination. However, the general preference for action (disregarding deontology and utilitarianism) is confused with utilitarian inclination; analogously, the general preference for inaction can be confused with deontological inclination. To untangle these confusions, MPT was first introduced into the analysis of moral judgment by Gawronski et al. (2017), who aimed to distinguish three independent parameters: (1) sensitivity to consequences in the utilitarian principle (*C*); (2) sensitivity to moral norms in the deontological principle (*N*); and (3) the general preference for inaction versus action without considering utilitarianism and deontology (*I*), namely, the consequences, norms, and generalized inaction (CNI) model. Although the CNI model excludes the influence of the general preference for inaction versus action, due to the limitation of the CNI model, the PD method is still required for correlation analysis.

Alterations in the moral judgment of individuals with substance use disorders have been found in previous studies, with alcohol‐dependent individuals (Carmona‐Perera et al., 2014) and polysubstance‐dependent individuals (Carmona‐Perera et al., 2012) showing more utilitarian judgment. At present, no research has assessed the responses of patients with IA to moral dilemmas, and the relationship between moral judgment and IA needs to be explored. Furthermore, the sensitivity to consequences, the sensitivity to moral norms, and the general preference for inaction versus action are not clear in patients with IA. Therefore, through traditional analysis, PD analysis, and the CNI model, the explicit moral judgment and implicit moral inclinations are comprehensively revealed in patients with IA. A great deal of research has confirmed that emotion plays an important role in moral judgment. For example, using PD analysis, researchers found that deontological inclination was positively correlated with empathic concern but negatively correlated with psychopathy (Conway & Gawronski, 2013; Reynolds & Conway, 2018). The CNI model further revealed that individuals with high levels of psychopathy showed a weaker sensitivity to moral norms, while those high in empathic concern were more sensitive to moral norms (Gawronski, Armstrong, Conway et al., 2017; Körner et al., 2020; Luke & Gawronski, 2021). We hypothesized that patients with IA, are similar to those with substance use disorders, would make more utilitarian judgment. For alcohol‐dependent individuals, it has been found that the impairment of emotion process is related to more utilitarian judgment (Carmona‐Perera et al., 2014). Therefore, we also have reason to believe that patients with IA make more utilitarian judgment because of the decline in deontological inclination or sensitivity to moral rules.

## 2 MATERIALS AND METHODS

### 2.1 Participants

More than 200 participants were recruited initially. All participants were first assessed by Internet Addiction Test (described in detail below), using total scores of ≤40 versus ≥50 to determine healthy control (HC) group versus IA group. Participants who meet the score criteria need to complete the next tasks, but the rest did not. Finally, a total of 40 healthy participants and 89 participants with IA were recruited. All participants reported that they had no major diseases and no history of mental diseases. Using the Fagerström Test for Nicotine Dependence (FTND) and the Alcohol Use Disorders Identification Test (AUDIT; described in detail below), none of the participants had alcohol, nicotine dependence. This study received approval by an ethical review committee in the Jiangsu Vocational College of Medicine (No. 202002). All participants provided signed consent and volunteered to participate in this study after understanding the tasks of the experiment. At the end of the experiment, all participants received a monetary reward. A participant in the HC group was distracted while performing a task, and another participant's data were damaged. The resulting usable data came from 38 normal Internet users, including 13 males and 25 females. Eighty‐nine IA participants, including 31 males and 58 females, were included. Demographic variables such as age, years of education, and Internet Addiction Test score are shown in Table 1. There was no significant difference between the two groups in gender, FTND score, and AUDIT score.

**TABLE 1:** Demographics of participants

	Control	IA			
	(*N* = 38)	(*N* = 89)	$t/\chi^{2}$	p	Cohen's *d*
Gender (male)	13	31	0.01	.95	/
Age	19.09 ± 0.74	19.17 ± 0.73	−0.57	.57	0.11
Education	13.32 ± 1.65	13.03 ± 1.12	1.12	.27	0.21
YIAT	28.18 ± 4.39	59.12 ± 7.41	−29.19	<.001	5.08
FTND	0.03 ± 0.16	0.06 ± 0.32	−0.57	.57	0.12
AUDIT	0.97 ± 2.00	0.75 ± 1.61	−0.66	.51	0.12

### 2.2 Materials

#### 2.2.1 Assessments

In this study, the Chinese version of Young's Internet Addiction Test (YIAT) (Young, 1998) was used to evaluate the presence and severity of IA. There are 20 items in the scale, and each item is scored on a Likert scale (1 = never, 5 = often). The overall score ranges from 20 to 100, with higher scores indicating serious Internet use problems. YIAT has been proven to have good psychometric characteristics in many countries, including China (Lai et al., 2013; Young, 2013). In general, a score less than 40 is considered normal Internet use, a score between 40 and 69 is considered a normal life problem due to excessive Internet use, and a score above 70 is considered a serious Internet use problem (Lai et al., 2013). Some studies have classified an overall score of more than 50 as IA (Chen et al., 2016; Sun et al., 2019; Wang et al., 2011). Combined with previous studies, to increase the differences between the two groups, this study defined participants with IA as those with a score greater than or equal to 50 and HC participants as those with a score less than or equal to 40. The Cronbach's *α* coefficient of the scale in this study was .94.

Nicotine dependence was assessed using the FTND. The score of ≤3 was determined as low dependence and ≥6 was determined as highly dependent (Ríos‐Bedoya et al., 2008). Alcohol dependence was assessed using the AUDIT, and total scores of 8 or more are generally recommended as possible alcohol dependence (Reinert & Allen, 2002).

### 2.3 Moral judgment task

The moral judgment task (Gawronski, Armstrong, Conway et al., 2017) consisted of six moral dilemma stories, namely, the abduction dilemma, transplant dilemma, torture dilemma, assisted suicide dilemma, immune dilemma, and vaccine dilemma. Each story was divided into two versions: the dilemma of proscriptive norms and the dilemma of prescriptive norms. In the former, action was prohibited by moral norms, but in the latter, action was prescribed by moral norms. Additionally, the two versions of the dilemma contain two types: the benefits of action are greater than the costs, and the benefits of action are smaller than the costs. Thus, the general preference for inaction is separated from the sensitivity to the norms (*N*) by manipulating the norm (proscriptive norm, prescriptive norm), and the general preference for action is separated from the sensitivity to consequences (*C*) by manipulating the consequences (benefits of action greater than the costs, benefits of action smaller than the costs). A total of 24 scenarios were used in the CNI model. Participants had to read each version one by one and then choose or refuse to take a given action.

#### 2.3.1 Procedure

Participants who met the inclusion criteria completed the moral judgment task. Twenty‐four moral dilemmas were presented separately on the computer screen using E‐prime software (provided by PST), and participants had to accept (press the F key) or refuse (press the J key) a specific action (without time limits imposed). After making a judgment, participants were asked to rate how difficult it was to make the judgment on a Likert scale of 1–5 (1 = very easy, 5 = very difficult). According to a previous study, all moral situations were presented in a fixed random order (Gawronski, Armstrong, Conway et al., 2017).

#### 2.3.2 Analyses

For each moral dilemma, the acceptance of an action was coded 1, and the refusal of an action was coded as 0. In traditional analysis, the number of utilitarian choices (acceptance of a particular action) is the dependent variable. In the PD analysis, two versions of proscriptive norms were used: benefits greater than the costs (incongruent; for example, sacrificing one person saves five people) and benefits less than the costs (congruent; for example, sacrificing one person for the health of another) (Conway & Gawronski, 2013). The deontological inclination (*D* parameter) and utilitarian inclination (*U* parameter) were the dependent variables. For analysis of the CNI model, all versions of the moral situation were analyzed, and the fitting degree and parameters of the model were compared. SPSS 24.0 was used for traditional analysis, PD analysis, and the difficulty of decision‐making analysis. The multiTree v0.47 program (Moshagen, 2010) and the multiTree template file for the CNI model (Gawronski, Armstrong, Conway et al., 2017) were used for CNI model analysis. A significance level of α<.05 was selected.

## 3 RESULTS

The mean and 95% confidence interval (CI) for acceptance of a specific action in each version of the moral dilemma for the IA group and HC group were shown in Table 2. Because there are six moral dilemma stories, the total score for each version is between 0 and 6. The preference for action or inaction can be determined by comparing the score with the neutral reference score of 3.

**TABLE 2:** The specific action in each version of the moral dilemma for the IA group and HC group

	Proscriptive norm prohibits action				Prescriptive norm prescribes action			
	Benefits of action greater than costs		Benefits of action smaller than costs		Benefits of action greater than costs		Benefits of action smaller than costs	
Group	*M*	95% CI	*M*	95% CI	*M*	95% CI	*M*	95% CI
Control	2.29	[1.83, 2.75]	1.82	[1.30, 2.33]	4.34	[3.99, 4.69]	3.74	[3.29, 4.18]
IA	3.24	[2.95, 3.52]	2.26	[1.95, 2.56]	4.09	[3.81, 4.37]	3.46	[3.20, 3.72]

*Note*: Moral dilemma judgment scores range from 0 to 6. The neutral reference score of 3. *M* is the mean. CI is the confidence interval.

### 3.1 Traditional analysis

Traditional moral dilemmas consider only one of the four versions of moral dilemmas used in this study, in which actions prohibited by moral norms and the benefits of the action to overall well‐being exceed the costs. In this version, acceptance of an action can be interpreted as a utilitarian judgment, and conversely, refusal of an action serves as a deontological judgment. The utilitarian judgment preference of IA group and HC group was analyzed in turn using a one sample *t*‐test. The results revealed no significant difference between the score of the IA group and the neutral overall preference score of 3, $t\;\left(88\right)=1.64,p=.104,\mathrm{Cohe}n^{{}^{\prime }}s\;d=0.35$, but the score of the HC group was significantly smaller than 3,$\;t\;\left(37\right)=-3.14,p=.003,\mathrm{Cohe}n^{{}^{\prime }}s\;d=1.03$. Furthermore, an independent sample *t*‐test revealed that the utilitarian preference of the IA group was significantly higher than that of the HC group$\;t\;\left(125\right)=-3.57,p=.001,\mathrm{Cohe}n^{{}^{\prime }}s\;d=0.69$. The IA group tended to be more utilitarian in their moral judgment than the HC group (Table 2).

### 3.2 PD analysis

**FIGURE 1:**
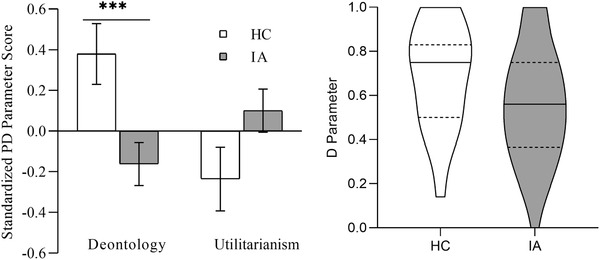
(Left) Mean standardized process dissociation (PD) deontology and utilitarianism scores for the IA group and HC group. Error bars depict standard errors. ***p value <.001. (Right) Violin plot of the *D* parameters of the HC group and IA group. Solid lines represent medians, and dotted lines represent quartiles

The PD involves only two versions of proscriptive norms: the benefits of an action are greater than the costs (incongruent), and the benefits of an action are smaller than the costs (congruent). The *U* and *D* parameters of each participant were calculated and standardized for further analysis (Conway & Gawronski, 2013). A 2 (IA/HC group) × 2 (*U/D* parameter) repeated‐measures ANOVA (see Figure 1) showed a significant interaction effect between group and parameter, $F\;\left(1,\;125\right)=12.22,\;p=.001,\;\;\eta_{p}^{2}=.09$. Furthermore, simple effect analysis showed no significant difference in the *U* parameter between the IA (M=0.10,SD=1.00) and HC groups (M=−0.24,SD=0.96), $F\;\left(1,\;125\right)=3.02,\;p=.08,\;\;\eta_{p}^{2}=0.06$, while the *D* parameter of the IA group (M=−0.16,SD=0.99) was significantly lower HC group (M=0.38, SD=0.92), F(1,125)=8.24, p=.005, $\eta_{p}^{2}=0.10$. Spearman's rho correlations revealed that the YIAT score was significantly related to the *D* parameter, r=−0.33,p<.001, but the correlation coefficient between the *U* parameter and YIAT score was not significant, r=0.14,p=.12 (see Table 3). These results showed that the increased utilitarian judgment in IA was related to the decreased deontological inclination rather than an increase in utilitarian inclination or a combination of the two possibilities.

**TABLE 3:** Pearson correlation coefficient between important variables (*N* = 127)

Variables	YIAT	Age	Education	Traditional score	*U* parameter
Age	.10				
Education	−.08	.30***			
Traditional score	.35***	.00	.04		
*U* parameter	.14	.03	−.06	.49***	
*D* parameter	−.33***	.02	−.04	−.78***	.09

*Note*: ***p value <.001.

### 3.3 CNI model analysis

The data of the two groups as a whole were entered into the multiTree program. The results showed the CNI model fitted the data well, $\Delta G^{2}\;\left(1\right)=0.951,p=.330$. Furthermore, the *C* parameter (M=0.120, 95% CI = [0.086,0.155]) was significantly higher than the baseline score of 0, $\Delta G^{2}\;\left(1\right)=46.665,p<.001$, and the same result was found for the *N* parameter (M=0.249, 95% CI = [0.209,0.288]), $\Delta G^{2}\;\left(1\right)=150.190,p<.001$. These results suggest that all the participants were highly sensitive to both consequences and moral norms in moral decision‐making. The *I* parameter (M=0.451, 95% CI = [0.425,0.477]) was significantly smaller than its neutral reference point of 0.5, $\Delta G^{2}\;\left(1\right)=13.647,p<.001$, which suggests that the participants tended to choose action over inaction.

**FIGURE 2:**
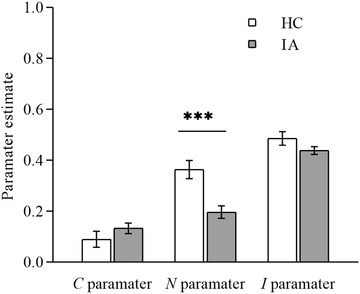
Parameter estimates of sensitivity to consequences (*C*), sensitivity to norms (*N*), and general preference for action versus inaction (*I*) for the IA group and HC group based on the multiTree program. Error bars depict 95% confidence intervals. ***p value <.001

The data of the IA group and HC group were also analyzed separately using the CNI model. The results showed that the model fit the data well (see Figure 2), $\Delta G^{2}\;\left(2\right)=2.207,p=.363$. For the IA group (M=0.197, 95% CI = [0.149,0.245]) and HC group (M=0.364, 95% CI = [0.294,0.434]), there was a significant difference in the *N* parameter, $\Delta G^{2}\;\left(2\right)=16.964,p<.001$. However, regarding the *C* parameter, no significant difference was observed between the IA group (M=0.133, 95% CI = [0.092,0.174]) and HC group (M=0.090, 95% CI = [0.028,0.151]), $\Delta G^{2}\;\left(2\right)=1.311,p=.252$. A similar result was found for the *I* parameter: no significant difference was observed between the IA group (M=0.438, 95% CI = [0.409,0.468]) and HC group (M=0.486, 95% CI = [0.434,0.539]), $\Delta G^{2}\;\left(2\right)=2.379,p=.123$. The above results indicate that the higher utilitarian judgment made by the IA group was related to a decrease in sensitivity to moral norms rather than an increase in sensitivity to consequences or a general preference for action.

### 3.4 Analysis of the difficulty of decision‐making

We also conducted a repeated‐measures ANOVA to assess the participants’ difficulty of decision‐making in the moral dilemmas. No significant group (IA/HC) × version of dilemma (congruent/incongruent) interaction emerged for difficulty of decision‐making, $F\;\left(1,\;125\right)=0.49,\;p=.485,\;\;\eta_{p}^{2}=0.004$. The main effect of group was significant for the subjective reported difficulty ratings, $F\;\left(1,\;125\right)=4.33,\;p=.039,\;\;\eta_{p}^{2}=0.03$, which revealed that the IA group reported more difficulty making a choice when presented with moral dilemmas than the HC group. For the dilemma version, the main effect was also significant, $F\;\left(1,\;125\right)=34.39,\;p<.001,\;\;\eta_{p}^{2}=0.22$. The result suggests that in the incongruent moral dilemma (the benefits of an action are greater than the costs), it was more difficult for participants to make choices than in the congruent moral dilemma (the benefits of an action are smaller than the costs).

## 4 DISCUSSION

This study was the first to explore the moral judgment of patients with IA. According to traditional analysis, patients with IA made more utilitarian judgment regarding sacrificial moral dilemmas than healthy Internet users. The PD analysis showed that there was no significant difference in utilitarian (*U*) parameter between the IA group and the HC group, while the deontological (*D*) parameter was significantly smaller in the HC group. Spearman's rho correlations revealed that the YIAT score was significantly related to the *D* parameter. The CNI model further showed that the sensitivity of IA to moral rules (*N*) was significantly lower than that of the HC group, while there was no significant difference between groups in the sensitivity to the consequence (*C*) and the general preference for action (*I*). In addition, the IA group reported greater difficulty than the HC group when they had to make a choice concerning moral dilemmas.

The results of traditional analysis showed that the explicit moral judgment of individuals with IA is similar to that of individuals with a substance use disorder. Although there is a lack of research on moral judgment of individuals with substance use disorders, previous studies have found some abnormalities in the moral judgment of individuals with alcohol (Carmona‐Perera et al., 2012; Carmona‐Perera et al., 2014; Khemiri et al., 2012) or opiate use disorders (Martin‐Contero et al., 2012). Alcohol‐dependent individuals were also found to be more utilitarian when faced with moral dilemmas than HC (Carmona‐Perera et al., 2014). In particular, there was a significant positive correlation between the severity of alcohol dependence and utilitarian judgment (Carmona‐Perera et al., 2012). Additionally, the researchers found that the ability of alcohol‐dependent individuals to decode emotions such as fear and pain was weakened, causing utilitarian judgment to increase (Carmona‐Perera et al., 2014). We considered whether the increase in utilitarian judgment of individuals with IA was also related to the abnormal emotion process. In the study of alcohol dependence mentioned above, only traditional analysis was used, so it was not possible to accurately account for changes in psychological inclinations (Carmona‐Perera et al., 2014). This finding may indicate an abnormality in the mechanism. In the current research, PD analysis and the CNI model were used to explain the change in psychological inclinations regarding explicit moral judgment.

The PD analysis revealed a decrease in deontological inclination among individuals with IA rather than an increase in utilitarian inclination, and the YIAT score was significantly related to the *D* parameter. According to the dual‐process model, individuals’ choices are driven by two independent and competing processes when they make a moral judgment: the emotion or intuitive process and the cognitive process (Greene, 2007). Deontological inclination is based on the emotional response to injury, and utilitarian inclination is based on cost–benefit analysis. The final moral judgment is determined by the relative strength of the two inclinations (Conway & Gawronski, 2013). Accordingly, these findings might indicate that when facing moral judgment, patients with IA have abnormal emotion process similar to those of alcohol‐dependent individuals. The PD analysis found a relationship between IA and abnormal moral judgment, but the precise influence of the general preference for action/inaction on individuals with IA must be clarified by the CNI model.

The current study used the CNI model to exclude the interference of general preference on action/inaction and further confirmed the findings of PD analysis. We observed a significant decrease only in the *N* parameter in the IA group. The general inclinations of individuals to ignore utilitarianism and deontology (for example, omission bias) can be confused with both moral inclinations (Crone & Laham, 2017; Gawronski, Armstrong, Conway et al., 2017). The CNI model approach clarifies this confusion by separating individuals’ sensitivity to consequences, sensitivity to rules, and general preference for action (Gawronski et al., 2018). This clarification further proved that the increase in utilitarian judgment among individuals with IA was related to a decrease in the sensitivity to norms, namely, deontological inclination. As noted, we can speculate that the increase in utilitarian judgment of individuals with IA is due to abnormalities in emotion process. In addition to the moral judgments of participants, the difficulty of decision‐making indicated by self‐reporting provides additional information.

In this study, the IA group experienced greater difficulty than the HC group when they made a choice in moral dilemmas. According to the dual‐process model, moral judgment depends on the relative strength between deontological inclination and utilitarian inclination. The closer both deontological inclination and utilitarian inclination were, the more conflict participants experienced in making judgment. Conversely, if one inclination was weaker than the other, it was relatively easy to make a given judgment (Conway & Gawronski, 2013). In current study, the HC group made more deontological decisions, suggesting that deontological inclination was stronger than utilitarian inclination. The PD analysis revealed that there was no significant difference in utilitarian inclination between the HC group and IA group, but the deontological inclination of IA group was less than that of HC group. Therefore, the relative strength between utilitarian inclination and utilitarian inclination in IA group was closer, and it is difficult to make a choice. Difficulty analysis further supports that sensitivity to norms decreased in the IA group. However, studies have found that in high‐conflict moral dilemmas, alcohol‐dependent individuals experience greater difficulty in decision‐making than healthy individuals (Carmona‐Perera et al., 2014). This difference may be attributed to the differences in moral materials and participants.

The relationship between some factors and moral judgment mentioned in the introduction seems to be similar to IA. For example, individuals with high levels of psychopathy showed a weaker sensitivity to norms, while higher empathic concern increases sensitivity of norms (Gawronski, Armstrong, Conway et al., 2017; Körner et al., 2020). Whether the changes of moral judgment of IA are related to these factors is worthy of further exploration. In addition, the early unpredictability may cause individuals to engage in more risks, opportunistic behaviors, and pursue immediate gratification later in life (Doom et al., 2016), which seems to be one of the related factors of IA. In terms of substance abuse, researchers have found that the early unpredictability is related to more substance use such as alcohol (Doom et al., 2016). A recent study also found unpredictability in childhood was associated with fewer deontological and utilitarian responses (Maranges et al., 2021). For the IA and the changes of moral dilemma judgment, the unpredictable childhood environment seems to play a source role, and the relationships between these factors are also worthy of further exploration.

Given the decrease in sensitivity to norms and the dual‐process model, the altered neural process of emotion and cognition may play an important role in this abnormal moral judgment in IA. Researchers have found abnormalities in the emotion system of patients with IA. Patients with IA show the change of functional connectivity in the amygdala (Cheng & Liu, 2020) and a reduced density of white matter in the inferior frontal gyrus, insula, amygdala, and anterior cingulate cortex (Ko et al., 2015; Lin et al., 2015). The amygdala and anterior cingulate cortex play an important role in the emotion process of moral judgment. The amygdala is responsible for processing social emotions and is particularly sensitive to the negative emotions induced by moral situations (Greene & Haidt, 2002; Shenhav & Greene, 2014). The anterior cingulate cortex is associated with conflict between cognitive and emotion process (Greene et al., 2004). Evidence in cocaine‐dependent subjects showed reduced activation in the ACC and left insula under moral judgment task functional magnetic resonance imaging (Verdejo‐Garcia et al., 2014). In addition, a study found patients with IA showed a higher primary somatosensorial cortex activation but a lower activation of limbic, temporal, and frontal area in response to emotional images, and this may be related to lower emotion involvement in emotional tasks (Lai et al., 2017). Further investigation should use network analysis to describe how brain processes conflict in moral dilemmas in a way that separates and integrates.

This study has some limitations. First, although the CNI model analysis can carefully separate the three parameters behind moral judgments, namely, sensitivity to consequence, sensitivity to norms, and general preference for action, this method is unsuitable for a design with more than one factor and correlation or regression analysis (Gawronski, Armstrong, Conway et al., 2017). Although some scholars added moral dilemma materials to make them applicable to the analysis of individual differences, they also increased the difficulty of the participants in completing the task (Körner et al., 2020). Therefore, the CNI model must be further optimized in the future. Second, although the moral dilemma materials used in this study have been improved in terms of their similarity to real life, they remain hypothetical situations. The ecological disinfection of materials for moral judgment must be further improved (Schein, 2020). Third, this study did not classify the subtypes of IA, so there may be different patterns of changes in moral judgments among individuals with different subtypes (e.g., Internet gaming addiction and social software addiction). Finally, the participants in this study were college students. Whether the conclusions can be generalized to other groups should be explored in future research.

In conclusion, the current work is the first to explore the performance of patients with IA in moral dilemma task, revealing the relationship between IA and moral judgment. Compared with the HC group, the IA group made more utilitarian judgments when faced with a moral dilemma. The results of PD analysis and the CNI model suggested that this increase can be attributed to decreased sensitivity to moral norms in individuals with IA rather than sensitivity to the consequence as well as a general preference for action. Future research should explore altered neuromachines for moral judgment in patients with IA.

## CONFLICT OF INTEREST

The authors declare that the research was conducted in the absence of any commercial or financial relationships that could be construed as a potential conflict of interest.

## AUTHOR CONTRIBUTIONS

L.‐J. X. and X.‐J. J. conceived and designed the research and wrote the original manuscript. C. J. and Z. Y. collected the data and participated in the literatures collecting and organizing. Z. H. provided guidance throughout the entire research process. J.‐Z. L. and W.‐Y. Q. helped translating and offered modification suggestions. All authors contributed to the article and approved the submitted version. J. X. and J. L. have contributed equally to this work and share first authorship.

### PEER REVIEW

The peer review history for this article is available at https://publons.com/publon/10.1002/brb3.2510

## Data Availability

The multiTree program (Moshagen, 2010) and the multiTree template file for the CNI model (Gawronski et al., 2017) were available free of charge on the Internet. All the data and results of this study are available at this link (https://osf.io/3vgwy).
